# Thermo-Oxidative Stability and Functional Properties of Extra Virgin Olive Oil Oleogels

**DOI:** 10.3390/gels12020116

**Published:** 2026-01-28

**Authors:** Denisse Bascuñan, Claudia Vergara, Cristian Valdes, Yaneris Mirabal, Roberto Quiroz, Jaime Ortiz-Viedma, Vicente Barros, Jaime Vargas, Marcos Flores

**Affiliations:** 1 Centro de Pomáceas, Universidad de Talca, Campus Lircay, Talca 3460000, Chile or denisseandrea.bascunan@rai.usc.es (D.B.);; 2 Department of Analytical Chemistry, Nutrition and Food Science, Food Technology Division, School of Veterinary Sciences, University of Santiago de Compostela, Campus Lugo, 27002 Lugo, Spain; 3 Departamento de Ciencias Básicas, Facultad de Ciencias, Universidad Santo Tomás, Talca 3460000, Chile; 4 Centro de Investigación de Estudios Avanzados del Maule, Vicerrectoría de Investigación y Postgrado, Universidad Católica del Maule, Avda. San Miguel 3605, Talca 3466706, Chile; 5 Institute of Applied Sciences, Faculty of Engineering, Universidad Autónoma de Chile, 5 Poniente 1670, Talca 3460000, Chile; 6 Campus Lircay, Universidad de Talca, Talca 3460000, Chile; 7 Departamento de Ciencia de los Alimentos y Tecnología Química, Facultad de Ciencias Químicas y Farmacéuticas, Universidad de Chile, Casilla 233, Santiago, Chile; 8 Departamento de Horticultura, Facultad de Ciencias Agrarias, Universidad de Talca, Campus Lircay, Talca 3460000, Chile

**Keywords:** oleogels, beeswax, thermo-oxidative stability, extra virgin olive oil, structural properties

## Abstract

Structuring oils using oleogels (OGs) represents a promising strategy for developing semi-solid lipid matrices with applications in food and other soft systems. This study evaluated the thermal stability and physicochemical properties of an oleogel (OG) formulated with extra virgin olive oil (EVOO) and beeswax (BW, 6%). The oleogel and olive oil samples were initially characterized by thermogravimetric analysis (TGA/DTG). The beeswax and oleogel samples were initially characterized by texture analysis. An antioxidant capacity (ORAC) analysis was initially applied to the beeswax sample. An initial rheometric analysis was applied to the oleogel sample. Fatty acid profiling and infrared spectroscopy were applied initially and finally to the oleogel and olive oil samples. During the thermal processing (80 °C, 14 days) of the oleogel and olive oil, analyses of the percentage of polar compounds, refractive index, and absorption parameters (K_232_ and K_270_) were performed. The oleogel exhibited a soft, pseudoplastic network, with lower hardness and mechanical strength than pure beeswax. Gelation modified the thermo-oxidative stability of EVOO, showing lower levels of polar compounds (from day 7 of heating; *p* = 0.028) and a slight delay in the onset of thermal degradation (Tonset), suggesting partial protection against the formation of polar degradation compounds. Furthermore, the evolution of K_232_ indicated differences in the formation of primary oxidation products (*p* = 0.027) over the 14 days of heating, while K_270_ showed no differences in the formation of secondary oxidation compounds. This reflects the complex interaction between the gelled matrix and the lipid deterioration mechanisms. Overall, the results demonstrate that the incorporation of beeswax allows for a partial reduction in degradation compounds in high-temperature processes, producing technologically functional oleogels that offer a potential alternative source for structuring solid fats. This work provides relevant evidence for the rational design of oleogels based on unrefined oils and opens new opportunities for their application in food systems and gelled matrices with thermal processing requirements.

## 1. Introduction

Lipid protection in foods is essential because these components are highly susceptible to oxidation, a degradative process that alters nutritionally valuable molecules and generates undesirable compounds responsible for rancidity, sensory defects, and loss of nutritional quality. Lipid oxidation also compromises product safety and shelf life. Therefore, the development of effective protection strategies such as natural antioxidants, advanced packaging technologies, or protective matrices is crucial to delay oxidative deterioration [1,2,3].

Extra virgin olive oil (EVOO), a hallmark of the Mediterranean diet, is globally recognized for its nutritional and functional value. In addition to being a dietary fat, EVOO contains a wide range of unsaponifiable compounds associated with health benefits related to cancer, chronic diseases, cardiovascular diseases, neurodegenerative diseases, and metabolic syndrome [4,5,6]. However, its stability under thermal conditions is limited since the presence of double bonds makes it highly susceptible to oxidation processes, enhanced by polyunsaturated fatty acids (PUFA) and, to a lesser extent, monounsaturated fatty acids (MUFA), accelerated by the exposure of light and oxygen [7,8].

Several approaches have been proposed to improve the thermo-oxidative stability of vegetable oils, including the use of leaf extracts from native South American trees as additives to improve the thermal behavior of avocado oil [9], the use of extracts from culinary ingredients such as clove and merkén chili to protect a polyunsaturated oil such as pumpkin seed oil during heating [10], the use of Tunisian pomegranate peel extracts to reduce the deterioration of corn oil subjected to high temperatures [11], and the use of green tea extracts to improve the oxidative stability and shelf life of sunflower oil during prolonged storage with performance comparable to synthetic antioxidants [12].

Oleogels (OGs) are innovative systems capable of structuring oils into semi-solid matrices without the need for hydrogenation. Several gelling agents have been documented for oleogel production, including low-molecular-weight compounds such as stearic acid and high-molecular-weight polymeric agents like plant waxes [13,14]. Among these, rice bran wax, sunflower wax, candelilla wax, and beeswax stand out due to their accessibility, low cost, and high gelation efficiency [15]. Oleogel formation is influenced by parameters such as temperature, the molecular weight of the gelling agent, oil composition, minor components, surfactants, concentration level, and processing conditions [16,17].

Recent applications of oleogels include, with the aim of reducing trans fatty acids and other nutritionally undesirable lipids. For instance, mixtures of corn and sunflower oils structured with beeswax have been employed in cookies, resulting in lower trans fat content and extended shelf life compared to liquid oils.

Beyond margarine applications, oleogels based on fish oil have also been formulated using rice bran wax and fatty acids as gelators, to develop foods with controlled-release properties. Another relevant application involves replacement of cocoa butter with oleogels formulated from transesterified amaranth oil. Furthermore, structured vegetable oils have been used to substitute animal fats in meat products, such as hamburger, reducing saturated fatty acids (SFA) while increasing PUFA content, particularly linolenic acid [18,19,20,21,22]. However, thermal processes remain a critical concern, as they involve high temperatures that promote degradative reactions capable of generating compounds potentially harmful to human health.

The use of unrefined vegetable oils, such as EVOO, in combination with natural oleogelators like beeswax has been widely explored to develop oleogels with functional and technological properties for foods and lipid matrices [23].

The development of oleogels from unrefined vegetable oils and structuring agents represents a promising strategy to enhance the stability, functionality and technological applications of fats. Evaluating their oxidative stability under prolonged heating provides essential insights into degradation mechanisms and physicochemical changes that occur during thermal processing, thus supporting the search for clean-label alternatives to synthetic additives.

## 2. Results and Discussion

### 2.1. Heating Treatment and Fatty Acid Profile

Table 1 shows the fatty acid profiles of the EVOO and OG samples before and after heat treatment. A slight increase in the proportion of SFA is evident after heating treatment in both samples. Palmitic acid (C16:0) increased significantly (*p* = 0.018) from 14.79% to 15.61% in EVOO and in the OG sample there was also a significant increase, rising from 14.40% to 15.95% (*p* = 0.014), while stearic acid (C18:0) exhibited a similar behavior with a significant increase (from 2.03% to 2.22% in EVOO and from 1.99% to 2.24% in OG, with *p*-values of 0.005 and 0.003, respectively). These changes could be attributed to the relative reduction in PUFAs, which are associated with lower thermal stability. A noteworthy case is of behenic acid (C21:0) in OG, which showed a very marked significant increase (from 0.09% to 0.43%, with *p*-value of <0.001), possibly influenced by the oleogel structuring agent.

Regarding oleic acid (C18:1 n9), the main component in both samples, its concentration remained stable and even showed a significant increase (EVOO: from 73.75% to 75.65%; OG from 74.07% to 75.41%, with *p*-values of 0.004 and 0.02, respectively). This behavior is associated with a high thermal stability of oleic acid, in agreement with what has been reported for unrefined vegetable oils rich in MUFA [7,9]. However, MUFAs present in lower proportions, such as eicosenoic acid (C20:1), showed greater susceptibility to degradation, decreasing significantly in both systems (EVOO: from 0.69% to 0.41%; OG: from 0.50% to 0.37%, with *p*-values of 0.004 and <0.001, respectively). Likewise, cis-10-heptadecenoic acid (C17:1) was significantly reduced by nearly 50% in OG (from 0.27% to 0.14%, with a *p*-value of 0.027).

For PUFAs, linoleic acid (C18:2 n6) was the most affected by heating, with significant reductions from 7.27% to 4.65% in EVOO and from 7.39% to 4.06% in OG, with *p*-values of 0.004 and <0.001, respectively. These results confirm the high susceptibility of PUFAs to thermal oxidation, even under moderate conditions (80 °C), where similar trends have been reported in other studies in which lipids are subjected to high temperatures [7,8], a phenomenon that leads to the formation of secondary oxidation compounds influence other analytical parameters. The magnitude of the loss was similar in EVOO and OG, suggesting that lipid structuring does not provide clear protection for PUFAs against oxidative degradation.

Overall, the behavior of the different fatty acid families was similar: a significant reduction in PUFAs, relative stability of oleic acid, and a slight increase in SFAs. However, the OG exhibited notable variations in the composition of minor fatty acids (e.g., behenic and heptadecenoic acids), possibly attributable to interaction between the vegetable oil and the gelling agent. Despite these differences, the post heating fatty acid profiles were comparable, confirming that the fatty acid composition of the oil is the main factor in thermal stability, while the OG structure provides partial protection.

### 2.2. Antioxidant Capacity

The antioxidant capacity (ORAC) measured for beeswax was 8718 ± 712.6 µmol of Trolox equivalents per 100 g, indicating that beeswax possesses moderate yet noteworthy, free radical scavenging activity. Although beeswax is not typically considered as a rich source of antioxidants compared with plant extracts rich in phenolic compounds (e.g., extracts from Veronica species or *Ugni molinae* fruit) [24,25], its crystalline structure and interaction with the oil phase have been reported to enhance the thermo-oxidative stability of vegetable oils during heating, as evidenced by studies on beeswax-based oleogels under accelerated oxidative conditions [26].

### 2.3. Total Polar Compounds Changes During Thermal Treatment

It is well known that beeswax has a complex composition, primarily composed of esters in a large proportion (>60%), followed by linear hydrocarbons (approximately 10%) and, to a lesser extent, long-chain alcohols, fatty acids, carotenoids, free acids, flavonoids, and a high proportion of the methylene group (-CH2, >95% of carbons present). This methylene group confers a hydrophobic character and good thermal stability, among other components that contribute to the formation of a semi-rigid structure with multiple functions, including microbiological, sensory, and physicochemical properties, allowing for its use as a structuring and thickening agent with good compatibility with lipids [a,b,c]. These results are preliminary regarding thermal stability and may serve as an approximation for the use of OG in high-temperature processes involving lipids of varying degrees of unsaturation.

**Figure 1:**
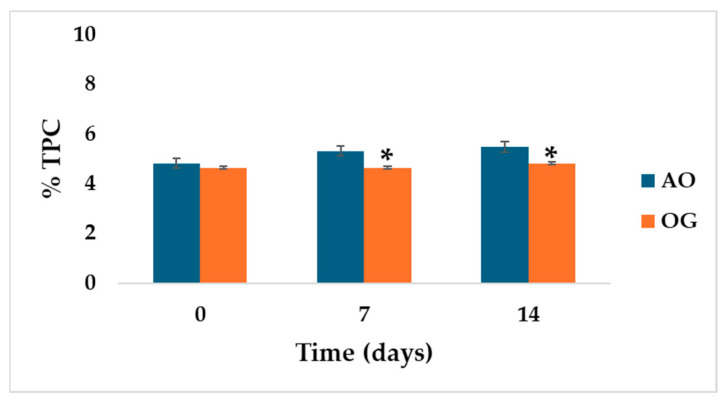
Evolution of total polar compounds (TPC) in extra virgin olive oil (EVOO) and oleogel (OG) samples during thermal treatment at 80 °C. * indicates a significant difference (*p* = 0.028).

According to Figure 1, the mean value of polar compounds plus its standard deviation (SD) showed significant differences (*p* = 0.028) for comparison of the sample and the control on day 14 of heating (5.50 ± 0.0% for EVOO and 4.83 ± 0.29% for OG). Total polar compounds (TPCs) represent a complex fraction of substances formed during thermal, oxidative, and hydrolytic degradation of oils, especially under frying conditions. This fraction includes peroxides, aldehydes, ketones, carboxylic acids, monoglycerides, diglycerides, free fatty acids, among others [27]. Because TPCs integrate multiple oxidation compounds, their quantification is considered a robust indicator of oil deterioration compared with individual parameters such as acidity index or peroxide value. Some international regulations and national guidelines establishes maximum limits for TPC in frying oils, typically ranging between 24 and 27%, highlighting their relevance for ensuring consumer safety [28,29]. These results are preliminary in terms of thermal stability and may be an approximation to the use of OG in high-temperature processes.

### 2.4. Acidity Index Changes During Thermal Treatment

In the case of the acidity index, only the samples from the third week of heating produced values between 0.07 ± 0.0.07 and 0.36 ± 0.50 (*p* = 0.21) above the instrument’s detection limit. However, these values were low (approximately 0.07%) and did not show significant differences between treatments. Previous studies have shown that the increase in acidity in vegetable oils during repeated heating and frying processes is generally slight and gradual, which is attributed to the fact that this parameter reflects the formation of only one type of degradation product [30].

### 2.5. Refractive Index (RI) Changes During Thermal Treatment

According to Table 2, both EVOO and OG showed a sustained maintenance of the RI throughout the heating period. When comparing the initial and final values of both samples, no significant differences were observed (*p*-values of 0.4 for EVOO and 0.3 for OG). However, during heating, significant differences were observed in the first two stages (*p* < 0.05), while no significant differences were found in the third stage (*p* = 0.12). In the case of OG, RI values were lower than those of EVOO.

The increase in this parameter is associated with the formation of oxidized compounds, polymers, and the degradation of fatty acids, mainly PUFAs, which increases the optical density of the oil [10].

It has been well established that RI increases significantly during high-temperature processes, such as frying, in both refined and unrefined vegetable oils [30,31]. This parameter is particularly relevant for assessing oil deterioration as it reflects the formation of degradation products such as ketones, aldehydes, and free fatty acids, whose formation rate depends on the initial lipid composition [32]. The lack of significant difference at the end of the heating period could be due to a change in the structured nature of the OG, attributable to the increase in oxidation processes, and the increase in both primary and secondary degradation compounds.

### 2.6. UV Absorption Coefficients (K_232_ and K_270_) Changes During Thermal Treatment

**Figure 2:**
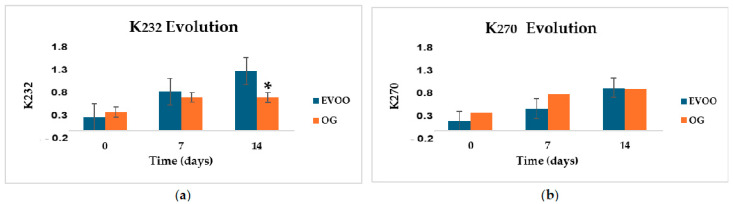
Evolution of UV absorption parameters (mean ± SD) of extra virgin olive oil (EVOO) and oleogel (OG) samples during thermal treatment at 80 °C: (**a**) K_232_; (**b**) K_270_. * indicates a significant difference (*p* = 0.027). n = 3.

The evolution of the UV absorption coefficients, is shown in Figure 2. For K_232,_ a significant differences between EVOO and OG was observed at day 14 (*p* = 0.027) with EVOO reaching higher values than OG samples. The K_232_ coefficient, associated with the formation of conjugated dienes and primary oxidation products, showed a progressive increase in EVOO throughout the 14-day heating period, whereas OG exhibited a more moderate and stable increase. This behavior suggests that the structural network of the oleogel limits the formation of primary oxidation compounds such as hydroperoxides, providing partial protection against primary oxidation. Similar trends have been observed when natural additives are added to vegetable oils to improve thermo-oxidative stability and effectively slowing the rise in K_232_ during high-temperature processing. For example, savory (*Satureja kitaibelii*) has been used to stabilize sunflower and olive oils [33], rosemary antioxidants have shown protective effects during deep frying in soybean oil [34], and saffron (*Crocus sativus*) stigmas has been valorized as a natural antioxidant for soybean oil stabilization [35]. In addition, culinary ingredients like “ají merkén” have been explored as protective components during thermal heating of edible oils [10].

On the other hand, K_270_, an indicator of secondary oxidation products, no statistically significant differences were observed between EVOO and OG throughout the heating period (*p* > 0.05), although both systems showed an increasing trend over time. Numerous studies report that K_270_ typically increases when lipids are exposed to pro-oxidant conditions for prolonged periods, regardless of whether the oils are enriched with antioxidant compounds, since the production rate of species absorbing at 270 nm varies with the treatment [35,36]. In our study, OG exhibited higher initial K_270_ values and a rapid increase during the first week, which may be attributed to absorbing compounds present in the gelling agent or to an early decomposition of hydroperoxides within the three-dimensional network. However, after 14 days, both systems reached similar values (≈1.0), indicating that the accumulation of secondary oxidation products tends to equalize over time. EVOO, in contrast, displayed a continuous increase across the entire heating period.

A comprehensive interpretation of these results requires considering both primary and secondary oxidation pathways. In this context, the oleogel matrix appears to inhibit the early formation of primary oxidation products (K_232_), while potentially facilitating, or not sufficiently delaying, the conversion of hydroperoxides into secondary species (K_270_). These findings underscore the importance of further investigating the role of gelling agents in lipid oxidative stability.

### 2.7. Thermogravimetric Analysis (TGA/DTG)

**Figure 3:**
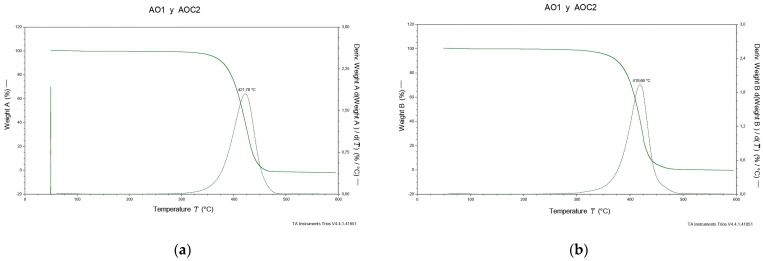
Thermogravimetric curves of (**a**) extra virgin olive oil (EVOO) and (**b**) oleogel (OG).

To evaluate the influence of structure and composition on the thermal degradation of EVOO and OG, thermogravimetric analyses were conducted over a temperature range of 20–600 °C. Thermogravimetric analysis, Figure 3, revealed differences in thermal stability between the two samples. Based on the tangent method, the onset temperature of thermal degradation (Tonset) was determined to be 397.3 °C for EVOO and 401.6 °C for OG, while the temperature of maximum degradation rate (Tmax) was approximately 425 °C for both systems. These results indicate that OG exhibits a slightly delayed onset of degradation compared with EVOO, reflecting greater thermal resistance during the initial decomposition stage. Additionally, the incorporation of natural extracts from various plant species has been reported to enhance the thermal behavior of oleogels [37].

Furthermore, complete degradation of EVOO occurred at 462.2 °C, whereas OG retained residual mass up to 552.1 °C. This behavior confirms that OG displays superior overall thermal stability, withstanding a broader temperature range prior to complete decomposition. Overall, the TGA-derived parameters indicate that the oleogel formulation performs more favorably under heating conditions, likely due to compositional differences and the stabilizing effect of beeswax. Forero-Doria et al. (2017) demonstrated that vegetable oils such as EVOO, when rich in total phenolics and MUFAs, tend to exhibit higher Tonset values in thermogravimetric analyses, reflecting greater resistance to deterioration processes [38].

### 2.8. Texture Profile Analysis (TPA)

Texture analysis showed a fractality value of 497 ± 4.5 g·s for the beeswax (BW) sample, indicating a rigid and brittle structure, whereas the oleogel (OG) exhibited a value of 0 g·s. Hardness was markedly higher in BW (7763 ± 45.6 g) compared with OG (79 ± 2.4 g) (*p* < 0.001). Similar behaviors have been reported in previously studied bigel systems [39].

BW also displayed considerably higher gumminess (1020 ± 17.8 g·s) and chewiness (420 ± 9.7 g·s), while OG showed much lower values (10 ± 1.2 g·s and 3 ± 0.6 g·s, respectively) determining *p*-values less than 0.001, indicating a material that readily disintegrates. BW exhibited greater elasticity (41.2% ± 1.4) and resilience (137.7% ± 3.1), characteristics associated with dense crystalline structures. In contrast, OG showed reduced elasticity (26.3% ± 1.4) and resilience (5.2% ± 0.3), reflecting a weaker structural network, with *p*-values of 0.004 and <0.001, respectively. The cohesiveness of both materials was comparable (BW: 13.1% ± 1.1; OG: 12.5% ± 0.7), suggesting similar resistance to internal fragmentation.

It is important to note that although OG consistently presented lower values than BW, the objective of structuring EVOO with BW was to obtain a semi-solid material. Comparable results have been previously reported, and it is known that the three-dimensional structure of such systems can be destabilized by additives that weaken intermolecular interactions [40].

### 2.9. Rheological Behavior

**Figure 4:**
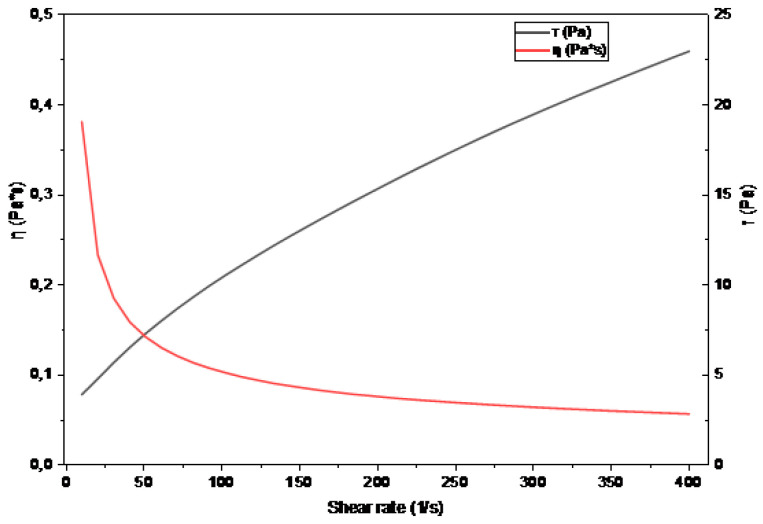
OG single-sample flow curves. The black line shows shear stress as a function of shear rate, and the red line shows apparent viscosity as a function of shear rate. Flow properties were evaluated over the range of 0.1–400 s^−1^.

Figure 4 shows the flow curves of the OG sample, illustrating the relationship between shear stress (τ) and shear rate (γ˙), as well as the variation in apparent viscosity (η). The material exhibited nonlinear behavior characteristic of non-Newtonian fluids, with viscosity decreasing progressively as shear rate increased. This shear-thinning behavior is associated with partial disruption of the oleogel’s structural network and the alignment of lipid molecules under mechanical stress [41].

The experimental data were fitted to several rheological models Newton, Bingham, Ostwald–de Waele, Casson (linear), and Herschel–Bulkley to describe the flow behavior of OG. The parameters and coefficients of determination (R^2^) are presented in Table 3.

The Herschel–Bulkley model provided the best fit (R^2^ = 0.9997), accurately describing the stress and shear rate relationship. In this model, the yield stress (τ_0_ = 1.90 ± 0.15 Pa) represents the minimum stress required to initiate flow, associated with the rupture of the three-dimensional network of wax crystals that immobilizes the oil within the matrix [42]. The consistency index (K = 0.415 ± 0.168) represents internal resistance to flow, while the flow index (n = 0.665 ± 0.031), being <1, confirms the oleogel’s pseudoplastic behavior. This type of behavior is typical of structured materials with an internal network that progressively disintegrates under stress, as previously reported in gelatin-based gels used to replace animal fat [43]. Similar model fits have been reported for the Herschel–Bulkley model in the formation of hydrogels based on polysaccharides and beta-glucans, with applications in food and biomedical applications [44].

The Bingham (R^2^ = 0.98) and Casson (R^2^ = 0.9963) models also provided acceptable fits but tended to overestimate yield stress and oversimplify low shear responses. The Ostwald–de Waele model (R^2^ = 0.9942) captured pseudoplasticity but did not account for yield stress, limiting applicability to materials with an internal solid structure. The Newtonian model (R^2^ = 0.76) was unsuitable because it assumes constant viscosity.

Shear stress response and pseudoplasticity are characteristic of dense crystalline structures with a three-dimensional network that governs flow behavior in oleogel systems. In comparison, the Bingham (R^2^ = 0.98) and Casson (R^2^ = 0.9963) models provided acceptable fits but overestimated yield stress and oversimplified low shear responses. The Ostwald–de Waele model (R^2^ = 0.9942) adequately represented pseudoplasticity, but did not account for yield stress, limiting its applicability to solid internal structured materials. Finally, the Newtonian model (R^2^ = 0.76) was unsuitable because it assumes constant viscosity, which does not reflect the behavior of the oleogel.

The rheological behavior of OG is advantageous for food applications because it confers stability at rest preventing syneresis or phase separation while shear-thinning facilitates processing and incorporation into semi-solid or emulsified matrices, improving texture and technological functionality [43,45].

### 2.10. Dynamic Rheology

**Figure 5:**
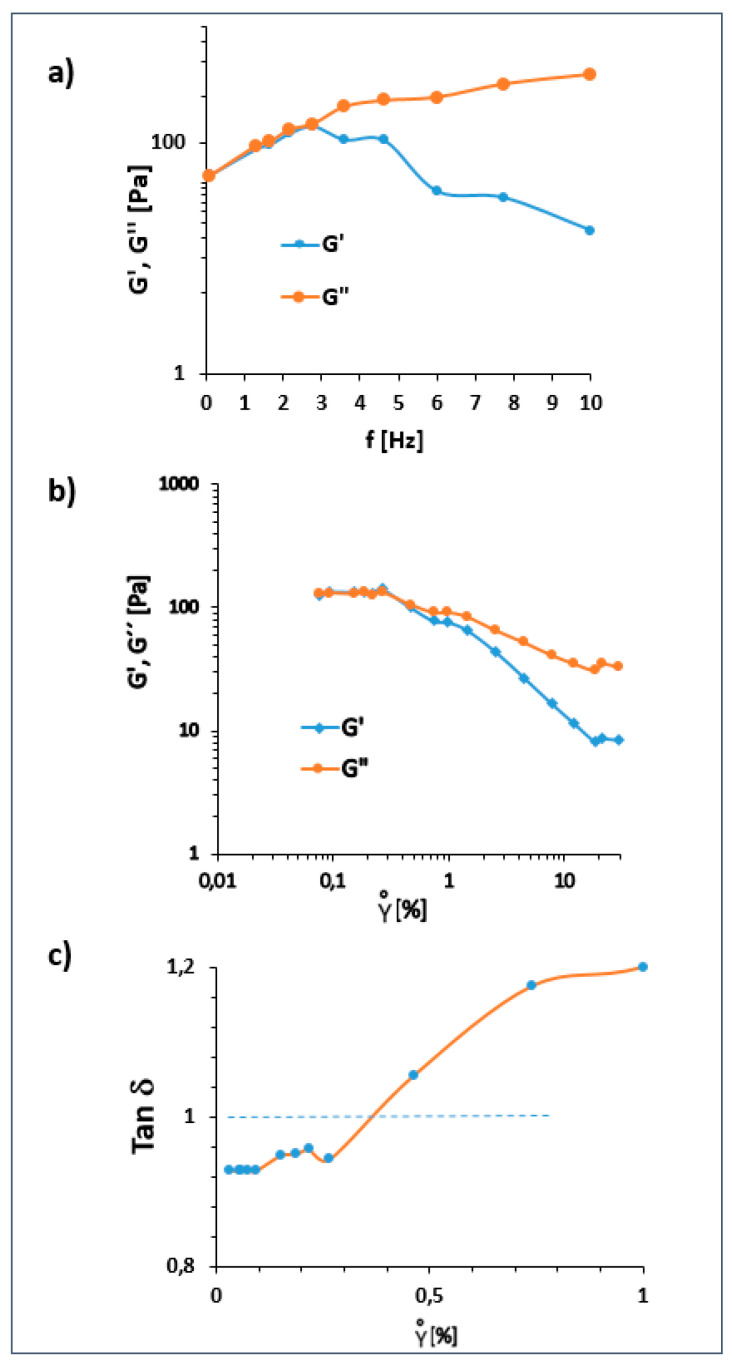
(**a**) Frequency sweep of the OG sample; (**b**) strain sweep showing the linear viscoelastic region and structural breakdown; (**c**) evolution of tan δ as a function of strain. Data obtained from a single sample.

The frequency sweep Figure 5a showed how the OG responds to oscillatory excitation at different angular frequencies. In the initial frequency region, both Storage Modulus (G′) and Loss Modulus (G″) increased with frequency; however, G″ dominated across most of the range, indicating a predominantly viscous response typical of weak gels. Fitting the complex modulus (G*) to the OG power law model (G* = k × f^n^) yielded k = 140.913 Pa·s^n^ and n = 0.358. The low value of n confirms that the material exhibits a soft and weakly frequency dependent structure, characteristic of physically structured oleogels.

The strain sweep Figure 5b revealed that the OG maintained a linear viscoelastic region (LVR) up to approximately 0.3% strain. Beyond this point, both G′ and G″ decreased, indicating progressive structural damage. Figure 5c shows the evolution of tan δ as a function of strain. At low strain levels (≈0.07–0.2%), G′ > G″ and tan δ < 1, confirming elastic-dominant behavior. At intermediate strains (0.2–1%), the moduli converged. For strains greater than 1%, tan δ exceeded 1, indicating that the material became more viscous as the network structure was disrupted.

The behavior observed is typical of oleogels structured by networks of crystalline lipid domains. Dominance of G′ at low strain confirms the presence of a self supporting three-dimensional network, whereas the rapid decay of both moduli with increasing strain suggests fragility, consistent with systems stabilized by weak van der Waals interactions or small lipid crystallites. The viscous dominance observed in the frequency sweep also indicates that the oleogel flows readily under dynamic stress, which aligns with functional attributes such as spreadability or controlled release [46].

Overall, the dynamic rheology results classify the OG as a weak gel with a short LVR, low structural robustness, and limited elastic recovery—characteristics typical of physically structured lipid networks. These properties make the OG suitable for food and cosmetic applications where spreadability, soft texture, and ease of deformation are desirable.

### 2.11. Fourier Transform Infrared Spectroscopy Analysis with Attenuated Total Reflectance (FTIR-ATR)

**Figure 6:**
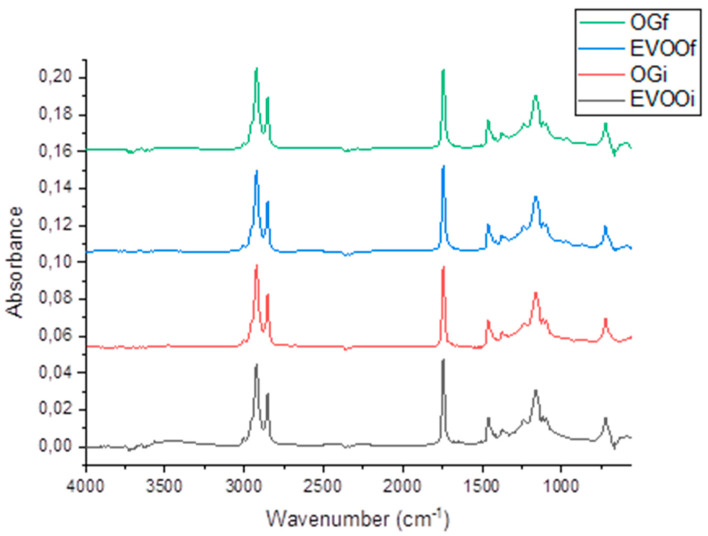
FTIR-ATR spectra of extra virgin olive oil (EVOO) and oleogel (OG) in their initial (i) and final (f) states.

Figure 6 presents the FTIR-ATR spectra of EVOOi and OGi. Both samples displayed characteristic triacylglycerol bands at ~2955 cm^−1^ (asymmetric aliphatic), 2853 cm^−1^ (symmetric aliphatic), and 1745 cm^−1^ (carbonyl stretching) [47]. EVOOi maintained a typical olive-oil spectral profile, whereas OGi showed slightly higher aliphatic band intensities and a slight reduction in the 3009 cm^−1^ unsaturation band, consistent with contributions from the gelling agent and increased structural ordering.

After heat treatment, both matrices exhibited an increase in the carbonyl band and a decrease in the vinyl band, indicating early oxidation. Both matrices exhibited similar spectral trends, suggesting that oleogel structuring does not markedly alter the loss of unsaturation during heating under the conditions tested [48,49].

## 3. Conclusions

This study shows that structuring extra virgin olive oil with beeswax produces an OG with modified mechanical, rheological, and thermo-oxidative properties relevant to the development of structured lipid systems. The OG presented a soft, cohesive, and pseudoplastic network, contrasting with the rigid and brittle behavior of beeswax. Dynamic rheological measurements confirmed that the material behaves as a weak physical gel, characterized by a short linear viscoelastic region, low frequency dependence, and a clear transition from elastic to viscous behavior once the structural limit is exceeded.

Thermogravimetric analysis and oxidation markers indicated that the OG provides partial protection during the early stages of thermal degradation, as reflected in a slight increase in Tonset and lower levels of polar compounds. However, the evolution of secondary oxidation indicators demonstrates that this protective effect is limited and may differ from that of the unstructured oil.

Overall, these results support the potential of beeswax-structured oleogels as functional lipid matrices suitable for food applications where controlled texture, spreadability, and improved thermal behavior are desired.

## 4. Materials and Methods

### 4.1. Oleogel Preparation

**Figure 7:**
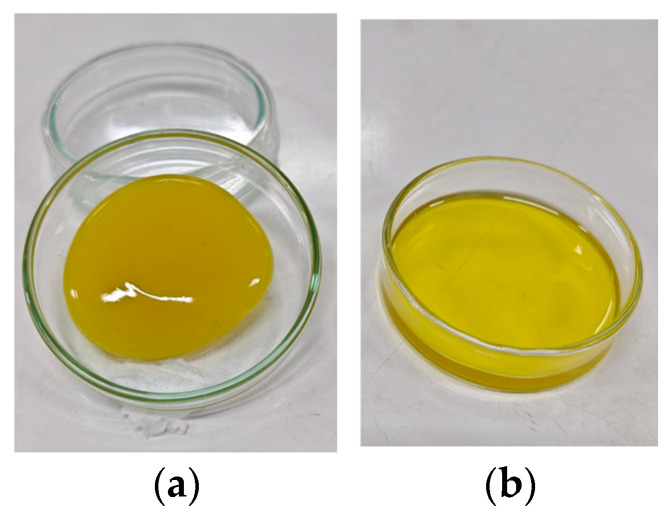
(**a**) Fresh oleogel (OG) 6% BW; (**b**) EVOO without heating.

Commercial extra virgin olive oil (EVOO) and beeswax (BW) obtained from the Maule region were used in this study. The oleogel (OG) was prepared by mixing EVOO with 6% BW under stirring (150 rpm) at 70 °C for 90 min with a Incubator Shaker model ES-20/60, Biosan (Riga, Latvia), following the optimized methodology proposed by Ankaraligil and Aydeniz Güneşer (2024) with slight modifications [49]. Most of the chemical analyses were applied to at least 3 separately prepared samples. Statistical analyses were performed using SPSS^®^ Statistics 19 software. Figure 7 shows the resulting OG.

### 4.2. Thermal Process, Experimental Design and Data Analysis

#### 4.2.1. Thermal Process

The thermal process consisted of constant heating of EVOO and OG samples at 80 °C in an oven without forced-air circulation for 14 days. These heating conditions have been based on the Schaal furnace test with slight modifications [50]. Parameters including total polar compounds (TPC), acidity index (AI), absorption coefficients (K_232_ and K_270_), and refractive index (RI) were measured weekly. Fatty acid profiles were determined by gas chromatography with flame ionization detection (GC-FID), while thermogravimetric analysis (TGA) and texture profile analysis (TPA) were also performed. Student’s *t*-test was applied to assess significant differences when appropriate.

#### 4.2.2. Fatty Acid Composition (GC-FID)

Fatty acids from EVOO and OG were transesterified into fatty acid methyl esters (FAMEs) using 0.2 N methanolic KOH, following Cert et al. (2000) with slight modifications [51]. FAMEs were analyzed using a gas chromatograph with flame ionization detector (GC-FID, EXPEC GC2000 model, Avenue, Qingshanhu Street, Lin’an District, Hangzhou, Zhejiang, China) equipped with a Restek Rt-2560 capillary column (100 m × 0.25 mm × 0.20 µm; Bellefonte, PA, USA, Restek Corporation). Nitrogen was used as carrier gas (1 mL/min; split ratio 1:20). Injector and detector temperatures were 240 °C. One microliter of each sample was injected.

The oven program was 70 °C to 180 °C at 3 °C/min, then 250 °C at 2 °C/min (15 min hold), based on Orsavova et al. (2015) with slight modifications [52].

Identification was performed using a certified FAME reference standard (Supelco 37 Component FAME Mix, Trace CERT^®^, Santiago, Chile). Quantification was based on area normalization. Each sample was derivatized and analyzed independently in triplicate (n = 3).

#### 4.2.3. Oxygen Radical Absorbance Capacity (ORAC) Method

The ORAC assay was performed according to the methodologies described by Huang et al. (2002) and Prior et al. (2003), with slight modifications [53,54]. The reagents used included Trolox, 2,2′-azobis(2-amidinopropane) dihydrochloride (AAPH), and sodium fluorescein (FL), all obtained from Sigma-Aldrich (Burlington, MA, USA). A 500 µM Trolox stock solution was prepared in 75 mM phosphate buffer (pH 7.4), and a calibration curve was constructed using Trolox concentrations ranging from 6.25 to 100 µM. Fluorescein (4 × 10^−6^ mM) was used as the fluorescent probe, while AAPH (150 mM) served as the peroxyl radical generator.

The assay was conducted in black 96-well flat-bottom polystyrene microplates (NUNC 237108, Roskilde, Denmark). Aliquots of 25 µL of either Trolox standards or appropriately diluted samples were added to each well, followed by 150 µL of fluorescein solution and 25 µL of AAPH. Measurements were performed at 37 °C using a Synergy HT microplate spectrofluorometer (BioTek, Winooski, VT, USA), with excitation and emission wavelengths set at 485 and 520 nm, respectively. Fluorescence decay was monitored for 60 min to determine antioxidant capacity.

#### 4.2.4. Total Polar Compounds and Acidity Index (AI)

The percentage of total polar compounds (TPC) and the acidity index (AI) were measured using a DOM-24 electrochemical sensor (Atago Co. Ltd., Minato-ku, Tokyo, Japan), operated according to the manufacturer’s instructions. Briefly, after each sampling from the oven, the sensor was immediately immersed in the lipid sample, and readings for both parameters were recorded once the instrument display had stabilized.

#### 4.2.5. Refractive Index

The refractive index of EVOO and OG was measured at 25 °C using a portable digital refractometer (Sper Scientific 300060, Scottsdale, AZ, USA). Prior to analysis, the instrument was cleaned and calibrated with distilled water. Subsequently, a few drops of the sample were placed on the prism surface, and the refractive index was recorded at 25 °C.

#### 4.2.6. UV Absorption Coefficients (K_232_ and K_270_)

The specific extinction coefficients at 232 nm (K_232_) and 270 nm (K_270_) were determined for the oil samples based on their absorbance at the corresponding wavelengths. UV measurements were performed using a UV/VIS spectrophotometer (Hanon i3, HANON Instruments, Jinan, Shandong Province, China) with 1 cm path-length quartz cuvettes, using hexane as the reference solvent. The extinction coefficients were calculated according to Equation (1):

(1) $$K_{\lambda }=\frac{D_{\lambda }}{C}$$

where K_λ_ is the specific extinction coefficient at wavelength λ, D_λ_ is the absorbance, and C is the oil concentration expressed in g/100 mL [55].

#### 4.2.7. Thermogravimetric Analysis

Thermogravimetric analysis (TGA) was performed to evaluate the thermal stability of the lipid samples, following the methodology described by Castro et al. (2021) with slight modifications [56]. Samples weighing between 10 and 20 mg were placed in platinum crucibles and heated from room temperature to 600 °C at a constant heating rate of 10 °C/min. Nitrogen (N_2_) was used as the protective gas at a flow rate of 50 mL/min to maintain the electronic balance.

In addition, derivative thermogravimetric (DTG) curves, corresponding to the first derivative of the TGA curves, were analyzed to determine the maximum decomposition temperature (T_max) of the samples. All analyses were performed using a Discovery SDT-Q650 thermogravimetric analyzer (TA Instruments, New Castle, DE, USA).

#### 4.2.8. Textural Characteristics

Texture profile analysis (TPA) was performed using an AGROSTA^®^ Belle texture analyzer (Serqueux, France) to evaluate beeswax and oleogel samples in terms of fracturability, adhesiveness, gumminess, chewiness, firmness, resilience, springiness, and cohesiveness. A conical probe (radius = 15 mm; height = 40 mm) was used to compress the samples under double-compression mode. The test parameters were set as follows: pre-test speed of 2 mm/s, test speed of 3.0 mm/s, and a 3 s interval between the first and second compression cycles. The trigger force was fixed at 0.2 N. All measurements were performed in triplicate.

#### 4.2.9. Rheological Measurements

##### Steady Shear Rheology

Steady shear flow curves were measured using a Thermo Scientific™ HAAKE RheoStress 1 controlled-stress rheometer (HAAKE GmbH, Karlsruhe, Germany) equipped with a plate–plate geometry (35 mm diameter). Samples were loaded between the parallel plates at 25 °C and adjusted to a gap of 1 mm. Steady shear tests were performed in controlled-rate mode by applying a linearly increasing shear rate from 0.1 to 400 s^−1^ over 1350 s at 20 ± 1 °C. All measurements were carried out in triplicate.

Experimental flow curves (shear stress versus shear rate) were fitted using the Herschel–Bulkley model (Equation (2)), commonly applied to describe shear-thinning fluids:

(2) $$\tau =\tau_{0}+k{\left(\gamma \right)}^{n}$$

where τ is the shear stress (Pa), τ_0_ is the yield stress (Pa), K is the consistency index (Pa·sⁿ), γ is the shear rate (s^−1^), and n is the flow behavior index. Data analysis was performed using HAAKE RheoWin Data Manager software (v.4.75, Thermo Scientific, Karlsruhe, Germany).

Dynamic rheological measurements were performed after allowing the samples to rest to ensure structural stabilization. Frequency sweep tests were conducted within the linear viscoelastic region (LVR) over a frequency range from 10 to 0.1 Hz in order to confirm that the viscoelastic response, expressed in terms of storage modulus (G′) and loss modulus (G″), was independent of strain amplitude. Based on these preliminary tests, a frequency of 1 Hz and a strain of 0.1% were selected for subsequent measurements.

The complex modulus (G*) was calculated from the frequency sweep data according to:

(3) $$G^{*}=\sqrt{{\left(G^{\prime }\right)}^{2\;}+{\left(G^{\prime \prime }\right)}^{2}}$$

The frequency dependence of G* was fitted to the weak-gel power-law model proposed by Gabriele et al. (2001) [57]:

(4) $$G^{*}=k\cdot f^{n}$$

where G* is the complex modulus (Pa), f is the frequency (Hz), k (Pa·sⁿ) is the strength parameter related to the interaction forces within the network (G* at 1 Hz), and n (dimensionless) is the coordination number or structural exponent, which reflects the extent of network connectivity [47].

Rheological data acquisition and modeling were carried out using RheoWin Job Manager 3 software (Haake/Thermo Fisher Scientific, Karlsruhe, Germany), which was employed to define the measurement protocols and experimental parameters, while RheoWin 3 Data Manager software (Haake/Thermo Fisher Scientific) was used for data processing, curve fitting, and modeling of the viscoelastic parameters.

Strain sweep tests were performed at a constant frequency of 1 Hz over a strain range from 0.1 to 1.0%, in order to determine the limits of the linear viscoelastic region and to evaluate structural breakdown under increasing deformation. All dynamic measurements were conducted at 25 ± 1 °C.

#### 4.2.10. Fourier-Transform Infrared Spectroscopy with Attenuated Total Reflectance (FTIR-ATR) of Initial and Final EVOO and OG

An FTIR spectrometer (FT/IR-4X model; Jasco Corporation, Hachioji, Japan) equipped with an attenuated total reflectance (ATR) accessory was used to record the spectra of EVOO and OG before (initial) and after (final) the heating process. Spectra were collected in the mid-infrared region (4000–550 cm^−1^), with a resolution of 4 cm^−1^ and 64 scans, following the methodology reported by Han et al. (2020) [47] with slight modifications. Spectra Manager 2.0 software was used for data acquisition and processing, while OriginPro 2019b software was employed for graphical representation. All measurements were performed at room temperature, using air as the background reference.

## Figures and Tables

**Table 1:** Fatty acid profile (%) of EVOO and OG samples before (i) and after (f) the 14-day heat treatment. Values are expressed as mean ± standard deviation (s.d.) (n = 3).

Fatty Acid	EVOO i (%)	EVOO f (%)	*p*-Value	OG i (%)	OG f (%)	*p*-Value
Myristic acid (C14:0)	0.012 ± <0.01	0.01 ± <0.01	0.069	0.01 ± <0.01	0.01 ± <0.01	0.02
Pentadecanoic acid (C15:0)	0.01 ± <0.01	0.01 ± <0.01	0.1	0.01 ± <0.01	0.02 ± 0.02	0.04
Palmitic acid (C16:0)	14.79 ± 0.08	15.61 ± 0.27	0.018	14.40 ± 0.43	15.95 ± 0.28	0.015
Palmitoleic acid (C16:1)	0.84 ± 0.01	0.84 ± 0.01	0.4	0.81 ± 0.05	0.84 ± 0.02	0.27
Heptadecanoic acid (C17:0)	0.14 ± 0.01	0.15 ± <0.01	0.055	0.15 ± <0.01	0.16 ± <0.01	0.06
cis-10-Heptadecenoic acid (C17:1)	^1^<D.L. 1	^1^<D.L. 1	-	0.27 ± 0.02	0.14 ± 0.06	0.03
Stearic acid (C18:0)	2.03 ± 0.03	2.22 ± 0.03	0.005	1.99 ± 0.04	2.24 ± 0.04	0.003
Oleic acid (C18:1 cis n9)	73.75 ± 0.14	75.65 ± 0.44	0.004	74.07 ± 0.37	75.41 ± 0.43	0.02
Linoleic acid (C18:2 trans n6)	7.27 ± 0.09	4.65 ± 0.34	0.004	7.39 ± 0.05	4.06 ± 0.10	<0.001
Arachidic acid (C20:0)	0.31 ± 0.02	0.35 ± 0.01	0.003	0.31 ± 0.01	0.37 ± 0.03	0.018
cis-11-Eicosenoic acid (C20:1)	0.69 ± 0.02	0.41 ± 0.03	0.004	0.50 ± 0.01	0.37 ± <0.01	<0.001
Behenic acid (C21:0)	0.15 ± 0.07	0.10 ± <0.01	0.16	0.09 ± 0.01	0.43 ± 0.02	<0.001

^1^ D.L. detection limit.

**Table 2:** Mean RI ± s.d values of EVOO and OG samples during heat treatment for 14 days at 80 °C. Data are expressed as mean ± SD of three independent measurements (n = 3). *p*-values indicate statistically significant differences between EVOO and OG at the same sampling time (*p* < 0.05).

Time (Days)	EVOO	OG	*p* Value
0	1.4674 ± 0.0013	1.4631 ± 0.0015	0.001
7	1.4667 ± 0.0021	1.4629 ± 0.0009	0.04
14	1.4676 ± 0.0002	1.4642 ± 0.0035	0.12

**Table 3:** Rheological parameters derived from steady shear flow curves using different models, along with their coefficients of determination (R^2^). Values are expressed as mean ± standard deviation (n = 3).

Model	τ0	Ƞp	ƞ	K	N	R^2^
Newton	-	-	0.067 ± 0.016	-	-	0.7634
Bingham	5.049 ± 0.562	0.048 ± 0.019	-	-	-	0.9800
Ostwald de Waele	-	-	-	0.832 ± 0.160	0.558 ± 0.054	0.9942
Casson (lin)	2.272 ± 0.216	0.029 ± 0.013	-	-	-	0.9963
Herschel–Bulkley	1.902 ± 0.148	-	-	0.415 ± 0.168	0.665 ± 0.031	0.9997

## Data Availability

The original contributions of the study are included in the article. Further inquiries can be directed to the corresponding authors.

## Abbreviations

The following abbreviations are used in this manuscript:

AI	Acidity Index
AAPH	2,2′-Azobis(2-amidinopropane) dihydrochloride
ATR	Attenuated Total Reflectance
BW	Beeswax
DTG	Derivative Thermogravimetry
EVOO	Extra Virgin Olive Oil
FAMEs	Fatty Acid Methyl Esters
FTIR	Fourier-Transform Infrared Spectroscopy
GC-FID	Gas Chromatography with Flame Ionization Detection
G′	Storage Modulus
G″	Loss Modulus
G*	Complex Modulus
K_232_	Specific Extinction Coefficient at 232 nm
K_270_	Specific Extinction Coefficient at 270 nm
LVR	Linear Viscoelastic Region
MUFA	Monounsaturated Fatty Acids
OG	Oleogel
ORAC	Oxygen Radical Absorbance Capacity
PUFA	Polyunsaturated Fatty Acids
RI	Refractive Index
SD	Standar deviation
SFA	Saturated Fatty Acids
TGA	Thermogravimetric Analysis
Tmax	Temperature of Maximum Degradation Rate
Tonset	Onset Temperature of Thermal Degradation
TPC	Total Polar Compounds
TPA	Texture Profile Analysis
UV	Ultraviolet
